# Tuning the Activity of a Hybrid Polymer–Oxocluster Catalyst: A Composition—Selectivity Correlation

**DOI:** 10.3390/polym13193268

**Published:** 2021-09-25

**Authors:** Giulia Bragaggia, Anna Beghetto, Ferdinando Bassato, Rudi Reichenbächer, Paolo Dolcet, Mauro Carraro, Silvia Gross

**Affiliations:** 1 Dipartimento di Scienze Chimiche, Università degli Studi di Padova, Via Marzolo 1, 35131 Padova, Italy; giulia.bragaggia@unipd.it (G.B.); annabeg93@gmail.com (A.B.); f.bassato@gmail.com (F.B.); rudi.reichenbaecher@tu-dresden.de (R.R.); paolo.dolcet@gmail.com (P.D.); 2 INSTM, Consorzio Interuniversitario per la Scienza e Tecnologia dei Materiali, Via Giusti 9, 50121 Firenze, Italy; 3 Fakultät Chemie und Lebensmittelchemie, Technische Universität Dresden, Helmholtzstr. 10, 01069 Dresden, Germany; 4 Institut für Technische Chemie und Polymerchemie, Karlsruher Institut für Technologie (KIT), Engesserstr. 20, 76131 Karlsruhe, Germany; 5 Istituto per la Tecnologia Delle Membrane del Consiglio Nazionale delle Ricerche (ITM-CNR), UOS di Padova, via Marzolo 1, 35131 Padova, Italy

**Keywords:** oxidation catalysis, hybrid materials, oxocluster

## Abstract

Zr-based oxoclusters M_x_O_y_(OR)_w_(OOR’)_z_ are promising catalysts for the activation of hydrogen peroxide. However, they need to be integrated into suitable matrices to increase their hydrolytic stability and allow for their recovery after use. Polymeric materials can be successfully employed for this aim, since they modify the properties of the resulting hybrid materials, in terms of polarity and chemical affinity for the substrates, improving the catalytic activity. Herein, we report the synthesis of different acrylic polymers based on various co-monomers (methyl methacrylate (MMA), 2,2,2-trifluoroethylmethacrylate (TFMA) and 3-methacryloxypropyltrimethoxylsilane (MAPTMS)) covalently cross-linked by a Zr_4_-based oxocluster, whose composition was tuned to optimise the catalytic oxidation of methyl p-tolyl sulphide. To assess their properties and stability, the materials were characterised via Fourier Transform Infrared (FT-IR) and Raman spectroscopies, Thermogravimetric Analysis (TGA), Solid-State NMR (SS-NMR) and X-Ray Absorption Spectroscopies XAS, before and after catalytic turnover.

## 1. Introduction

The preparation of hybrid materials integrating catalytic units represents a promising possibility to design heterogeneous catalysts [1]. Class II hybrid materials in particular are made of organic and inorganic components, held together by covalent bonds [2]. Among the available inorganic nanofillers, different authors reported the use of polyhedral oligomeric silsesquioxanes (POSS) [3,4], polyoxometalates (POM) [5,6,7] or oxoclusters of early transition metals [8]. Oxoclusters, with a general formula of M_y_O_x_(OH)_w_(O(O)CR)_z_, are a versatile class of polynuclear compounds including early transition metal ions M, such as Ti^IV^, Zr^IV^, Hf^IV^ or Nb^V^, linked by oxygen bridges and coordinated by organic ligands bearing bidentate, typically carboxylic, moieties [9,10]. Oxoclusters containing alkaline earth metals (e.g., Ba, Mg) as heteroatoms were also reported [11]. When the organic components of the hybrids are organic polymers/co-polymers, oxoclusters decorated with polymerisable groups can act as multi-functional cross-linking agents. As a result, several covalent bonds endow the final material with enhanced stability, preventing phase separation and leaching [12].

Since the oxoclusters are made of early transition metals in their highest oxidation state, they are appealing candidates for the catalytic activation of peroxides [13,14]. As far as Zr-based oxoclusters are concerned, we have recently evidenced the possibility to use different molecular hybrids to oxidise organic substrates (such as sulphur compounds) in the presence of hydrogen peroxide [15]. Once integrated into a polymethylmethacrylate (PMMA) matrix, the oxoclusters demonstrate improved hydrolytic stability, while the thermal stability of the polymer can also be increased [8,15]. Moreover, thanks to the enhanced affinity of the polymeric matrix towards polar substrates, highlighted by its swelling properties, the heterogeneous catalyst shows better catalytic performances than the corresponding homogeneous system. However, despite such notable advancements, the material still needs to be optimized in terms of stability and recyclability before becoming competitive with other heterogeneous systems.

In this paper, novel Zr-oxocluster (Zr_4_O_2_(OMc)_12_ (OMc = methacrylate)-based polymers were prepared by changing the nature of the monomers and the oxocluster/monomers molar ratio. Fluorinated or silane-containing monomers were used to evaluate the impact of the composition on the catalytic performance towards the oxidation of an organic sulphide (methyl p-tolyl sulphide) via H_2_O_2_ in an acetonitrile mixture [14], showing optimal performances of the catalysts in terms of stability, selectivity and activity. Concerning the reaction under investigation, the oxidation of sulphides has many applications for the preparation of pharmaceutical derivatives and is attracting growing interest as fuel desulphurisation strategy [16,17]. Moreover, acetonitrile is one of the polar solvents used to extract organic sulphides and their oxidised products from a fuel [18]. FT-IR, Raman, SS-NMR and XAS measurements were carried out on the pristine hybrids as well as on the materials after catalysis, to investigate their stability. As demonstrated by the associated kinetic study, the new polymers display tuneable reaction selectivity. The use of the fluorine-rich material springs in an excellent catalytic performance in terms of reaction yield, sulfone selectivity and recyclability, thus outperforming the former MMA–oxocluster hybrids.

## 2. Materials and Methods

Zirconium butoxide (Zr(OBu)_4_, 80% wt. in *n*-butanol), and methacrylic acid were employed for the synthesis of the oxocluster and were purchased from Sigma-Aldrich, while methyl methacrylate (MMA, 99% wt.), 2,2,2-trifluoroethylmethacrylate (TFMA, 99% wt.), 3-methacryloxypropyltrimethoxysilane (MAPTMS), benzoyl peroxide (≥97% wt.) and toluene, purchased from Sigma-Aldrich, were used for the synthesis of the hybrid materials. The monomers were previously filtered on neutral alumina to remove the inhibitors. Hydrogen peroxide (35% wt. in H_2_O), acetonitrile (≥99.8%), methyl p-tolyl sulphide (99% wt.), purchased from Sigma-Aldrich, were used for the oxidation reaction. Dichloromethane (≥99.9%), undecane (≥99%), triphenylphosphine, were used as solvents, a reference compound and an H_2_O_2_ quencher for the GC analysis, respectively.

### 2.1. Preparation of Zr_4_O_2_(OMc)_12_

Methacrylic acid was added to Zr(OBu)_4_, under argon, in a molar ratio alkoxide/methacrylic acid of 1:7, according to the literature procedures [19,20] and allowed to stand overnight. The mother liquor was decanted from crystals and then the crystals were dried under mild vacuum for 4 h.

### 2.2. Preparation of Hybrid Materials

In a typical polymerisation reaction implemented by Trimmel et al. [21], a weighted amount of oxocluster was dissolved in toluene (0.70 g of oxocluster per 4.0 g of toluene) and the monomers were added under gentle stirring, in a molar ratio of oxocluster to the sum of monomers of 1:50 or 1:100; the molar ratio between MMA and the co-monomer was varied from 9:1 to 5:5; benzoyl–peroxide (2% wt. with respect to the sum of the monomers), used as polymerisation initiator, was added when the temperature was at 85 °C and the reaction was carried out for 1 h under stirring. The obtained polymers were dried under vacuum at 60 °C for at least 3 h.

### 2.3. FT-IR Measurements

The products were characterized by FT-IR in transmittance mode by dispersing the sample in a KBr pellet, using a Thermo Quest Nicolet 5700 instrument from 4000 cm^−1^ to 400 cm^−1^ with 32 scans and a resolution of 4 cm^−1^.

### 2.4. Raman Measurements

Raman spectra were collected by using a Thermo DXR Raman Microscope (Department of Chemical Science, University of Padova, Padova, Italy). A 532 nm laser was used as excitation source, operating at 1–10 mW power with an exposure time of 5 s. The Raman mapping were collected on a region of 150 µm × 200 µm and a power of 8 mW.

### 2.5. Thermogravimetric Analysis (TGA)

The TGA were collected with a TGA Q500 TA Instruments with a ramp of 10 °C/min from room temperature (25 °C) up to 900 °C, under air flow.

### 2.6. Swelling Measurements

The swelling experiments were carried out by leaving a weighted amount of polymer in three different solvents for 72 h; after withdrawal from the solvent, the wet specimen was weighted again. The swelling index (*I_sw_*) was determined by the following formula:

(1) $$I_{sw}=\frac{\left(wt_{wet}-wt_{dry}\right)}{wt_{dry}}$$

### 2.7. Solid State NMR Spectroscopy

The solid state Nuclear Magnetic Resonance Spectroscopy analyses (SS-NMR) involved only the ^13^C nucleus; the spectra were acquired with a 400 MHz NMR Varian instrument (Department of Chemical Science, University of Padova, Padova, Italy) with a solid MAS probe and a spinning rate of 5.5 KHz. ^13^C Cross Polarization spectra were recorded with a linear shape, 2000 scans, contact time 1 ms, recycle delay 3 s, acquisition time 0.02 s, 800 points.

### 2.8. XAS Measurements

The XAS measurements were performed at the B18 beamline at Diamond Light Source (Didcot, UK). A Si (111) double crystal monochromator was used for measurements at the Zr K-edge (17.998 keV). The second monochromator crystal was tilted for optimal harmonic rejection. The spectra were recorded in transmission mode using ionization chambers as detectors. Energy calibration was performed with a Zr metal foil. The solid samples were pressed into self-supporting pellets using cellulose as a binder. Data evaluation started with background absorption removal from the experimental absorption spectrum by using the automated removal routine found in the Athena software [22]. The threshold energy E_0_ was determined as the maximum in the first derivative spectrum. To determine the smooth part of the spectrum corrected for pre-edge absorption, a piecewise polynomial was used. It was adjusted in such a way that the low-R components of the resulting Fourier transform were minimal. After division of the background–subtracted spectrum by its smooth part, the photon energy was converted to photoelectron wave numbers *k*. The resulting function was weighted with *k*_3_ and Fourier transformed using a Hanning window function. Data analysis was performed in k-space on unfiltered data, using the Artemis software 0.9.25.

### 2.9. GC Analysis

The solutions were analysed by Shimadzu GC2010 instrument equipped with an ionization flame detector and an Equity-5 (15 m × 0.1 mm) capillary column of poly(5% diphenyl/95% dimethylsiloxane) with 0.1 μm film thickness.

Operating conditions: T_inj_ = 270 °C; T_det_ = 280 °C; Carrier gas=He; Linear velocity = 40.0 cm/s; T_initial_ = 90 °C for 1 min; Ramp rate = 40 °C/min; T_final_ = 260 °C for 2 min.

### 2.10. Catalytic Tests and Catalytic Recycles

The oxidation reactions were carried out in a closed vial at 50 °C by dissolving a weighted amount of oxocluster in 1.2 mL or 2.2 mL of acetonitrile and 136 µL of methyl p-tolyl sulphide (1 mmol), to obtain a molar percent ratio oxocluster/sulphide of 0.28–0.29%. Then, 2 mmol of H_2_O_2_ (from a 35% wt. aqueous solution) were added to the solution under stirring. The reactions were monitored for 4 h. 50 µL aliquots of sample were withdrawn at fixed interval times and diluted in a 10 mM solution (1100 µL) of undecane in CH_2_Cl_2_ for GC analysis; excess triphenylphosphine, dispersed in the CH_2_Cl_2_ solution, was used to quench the residual peroxide.

Concerning the catalytic recycles, the catalyst was recovered, washed three times with 3 mL of acetonitrile and the reaction was repeated under the same diluted conditions for 4 h.

### 2.11. SEM-EDX Analysis

Field-emission scanning electron microscopy (FE-SEM) and energy-dispersive X-ray analysis (EDX) were run on a Zeiss SUPRA 40VP equipped with an Oxford INCA x-sight X-ray detector. Morphological analysis was carried out by setting the acceleration voltage at 5 kV, whereas the EDX compositional investigations were obtained by setting the acceleration voltage at 20 kV.

## 3. Results and Discussion

### 3.1. Synthesis and Characterization of the Hybrid Materials

**Figure 1:**
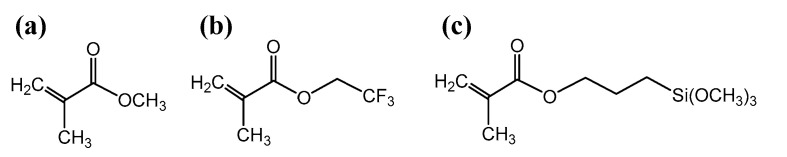
Monomers employed in the hybrid polymers synthesis: (**a**) methyl methacrylate (MMA); (**b**) 2,2,2-trifluoroethylmethacrylate (TFMA); (**c**) 3-methacryloxypropyl trimethoxysilane (MAPTMS).

Monolithic polymers were prepared by using, as starting monomers, methyl methacrylate (MMA), 3-methacryloxypropyl trimethoxysilane (MAPTMS) or 2,2,2-trifluoro ethylmethacrylate (TFMA) (Figure 1) [23,24,25].

MAPTMS was used as an organic–inorganic monomer to evaluate the effect of additional cross-linking via siloxane chains on polymer swelling/solubility and substrate selectivity. TFMA was instead used to evaluate the impact of oxidatively stable C-F bonds and of a fluorinated hydrophobic domain [26]. With the aim of monitoring the effect of different experimental parameters on the polymers’ properties, the nature of the monomers and their molar ratio were systematically changed (Table 1).

In particular, two oxocluster/monomers molar ratios (1:50 and 1:100) were explored to evaluate the effect of the oxocluster concentration and of the resulting cross-linking degree, on swelling and reactivity. On the other hand, the molar ratio of MMA to TFMA was tuned (from 9:1 to 5:5) to optimise polymers’ performances in terms of swelling, substrate selectivity and stability. The novel polymeric materials were compared with a previously prepared material containing only MMA in the same molar amount [14]. In Table 1, the experimental molar ratios used for the syntheses of the hybrids are reported in detail.

**Figure 2:**
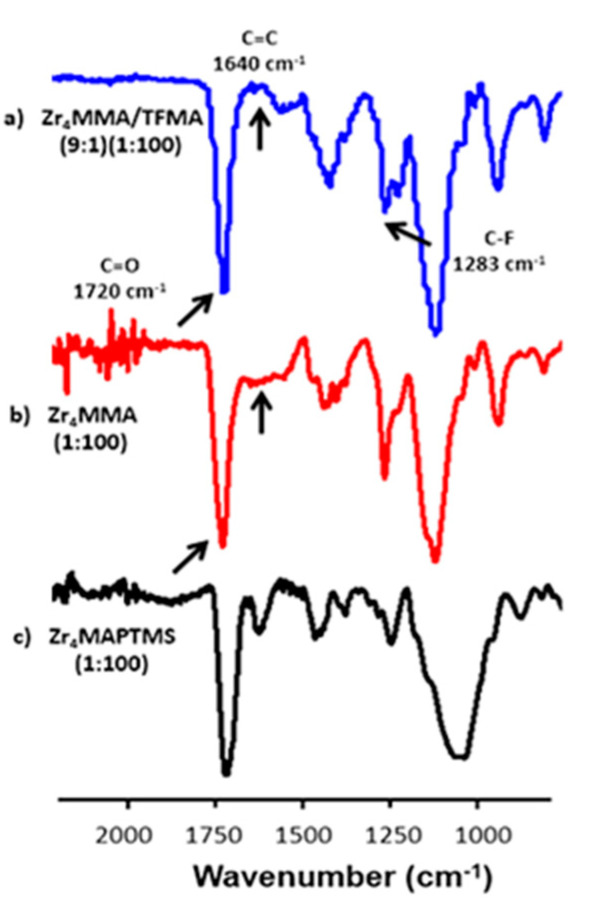
FT-IR spectra of samples: (**a**) Zr_4_MMA/TFMA (9:1) (1:100), (**b**) Zr_4_MMA (1:100) and (**c**) Zr_4_MAPTMS (1:100) in the region between 500 and 2500 cm^−1^.

Firstly, the materials were analysed by FT-IR analysis to specifically detect the presence of residual signals due to unreacted double bonds (Figure 2).

For a semi-quantitative evaluation of the degree of polymerisation, the intensity of the stretching signal around 1640 cm^−1^, related to C=C bonds, was compared to the stretching band around 1720 cm^−1^, related to C=O bonds. Indeed, the C=C/C=O intensity ratio is lower for higher polymerisation degrees [27]. In Figure 2, Figures S1 and S2, the most representative FTIR spectra are reported.

**Figure 3:**
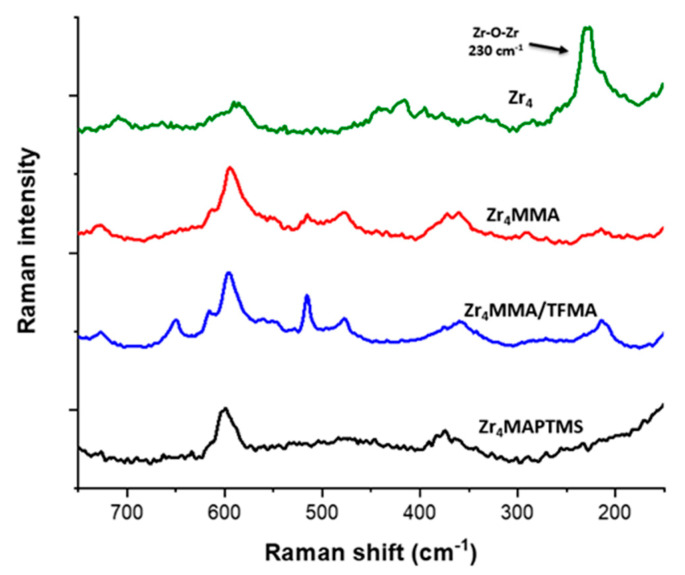
Raman measurements of Zr_4_-oxocluster, Zr_4_MMA and Zr_4_MMA/TFMA hybrids in the region between 150 and 750 cm^−1^.

In Figure S1, the peaks around 3000–2900 cm^−1^ of the MMA and TFMA-based samples are due to aliphatic C-H stretching [14]. The bands at 1463 cm^−1^and 1426 cm^−1^ are related to the bending of CH_3_ and the band at 1194 cm^−1^ corresponds to the asymmetric stretching of C–O–C [20]. Another meaningful signal is the peak around 1283 cm^−1^, related to the stretching of C-F bond, characteristic of TFMA-based copolymers [28]. The band related to C=C double bonds belonging to the oxocluster (as well as to the monomer) is found at 1640 cm^−1^. The Zr_4_MMA/TFMA samples show a negligible intensity of C=C signal in every sample. The very low C=C/C=O intensity ratio demonstrates a nearly complete radical polymerisation, even better than Zr_4_MMA/MMA, despite the steric hindrance of the oxocluster. Concerning the hybrid with MAPTMS (Figure S1 and Figure 2c), it is possible to observe a very broad band around 3460 cm^−1^, ascribed to the vibration of surface bound water molecules and to the silanol groups (which also give the band at 1640 cm^−1^) [29], thus highlighting a not completely condensed silica network [30,31]. The relatively weak absorption bands around 2945 cm^−1^ and 2840 cm^−1^ are attributed to the stretching of C–H bonds in alkyl and methoxy groups, respectively. The bands at 1717 cm^−1^ and 1638 cm^−1^ were assigned to the stretching of C=O and C=C groups, respectively; the one related to C=C shows a higher intensity with respect to the other polymers, revealing a less efficient radical polymerisation for this material. The broad band centred at 1080 cm^−1^ and the weaker band at 780 cm^−1^ confirm the presence of a Si–O–Si network, with its asymmetric and symmetric stretching, respectively [32]. Subsequently, in order to investigate the presence of the oxocluster in the synthesised hybrids, Raman measurements were carried out, since the stretching vibrations of Zr–O–Zr appear at low wavenumbers (<250 cm^−1^) and cannot be detected using conventional FTIR [15,33,34]. In Figure 3, the superimposition of the Raman spectra of the oxocluster and of the hybrid materials is reported.

The signal at 230 cm^−1^ is related to Zr–O–Zr bending modes [15]. It is worth noting that this feature is very intense in the spectrum of the Zr_4_ oxocluster sample, but it is much lower for the other samples, owing to the “dilution” of the oxocluster in the macromolecular host matrix. Nevertheless, a Raman mapping (Figure S4) was collected for the hybrid Zr_4_MMA (1:100) upon monitoring of the signal at 230 cm^−1^, showing a homogeneous distribution of the oxocluster into the polymer. Another peak, related to Zr–O stretching vibration, can be found at 598 cm^−1^; however, the latter appears overlapped by other signals belonging to the polymers. The homogeneous morphology as well as the regular elemental distribution of the samples were also confirmed by SEM (scanning electron microscopy) and EDX (energy dispersive X-ray analysis) of representative samples (Figures S9–S13). In all cases, bulky non-porous polymers, with no phase separation, were observed.

In order to determine the amount of inorganic domains in the hybrids [14] and to assess their thermal stability as a function of cluster content and co-polymer composition, the TGA of the most representative polymers were carried out under air (Figure S5). In all MMA and TFMA-based hybrids, it is possible to highlight a first relevant weight loss in the range 300–425 °C, which is ascribed to the degradation of the organic matrix of the samples. A second weight loss step, visible at a temperature around 450–510 °C, is related to the degradation of the organic fraction of the oxocluster. The improved thermal stability of these hybrids can be highlighted upon comparison with the TGA of the oxocluster-free MMA-based polymers (see Figure S5d,e), for which the main degradation starts already at 200 °C. Regarding Zr_4_MAPTMS(1:100), its weight loss starts at 400 °C, confirming its expected higher thermal stability arising from double cross-linking, while a second decomposition step is found between 450 and 580 °C, where the amount of the final residue is higher than for the other hybrids, owing to the relevant contribution of residual SiO_2_ derived from MAPTMS decomposition (up to 32% wt.). Since every oxocluster Zr_4_O_2_(OMc)_12_ produces 4 moles of ZrO_2_, relaying on its residual %wt., and under the assumption of homogeneity of the hybrids structure, it is possible to estimate the actual quantity of oxocluster embedded into the polymer. In Table 2, the observed residual weights % for the most representative hybrid materials (see the corresponding TGA in Figure S5f) are listed and compared with nominal values. The found values are in agreement with the theoretical ones, although the MMA-based polymer seems less efficient in terms of oxocluster incorporation.

Subsequently, in order to evaluate the affinity of the hybrids towards different solvents, swelling measurements were carried out. Swelling index can also provide a relative estimation of the cross-linking degree of the polymeric networks [35]. Swelling is due to two competitive phenomena: the increase of the whole solvent-solute entropy, due to the introduction of the solvent, and a decrease of polymeric chains entropy because of isotropic expansion [36]. In Table 3, swelling data of the hybrids materials in four different solvents, ethyl acetate (EtOAc), acetonitrile (ACN), ethanol (EtOH) and water (H_2_O), characterised by increasing polarity, are listed.

It is reported that solvent polarity parameter E_T_(30), based on the exceptional negative solvatochromism of 2,6-diphenyl-4-(2,4,6-triphenylpyridinium-1-yl) phenolate (denoted as betaine 30) [37,38] is a good descriptor of non-covalent interactions with solvents. Therefore, it is more suitable than dielectric constant or dipole moments to assess the polarity of different solvents.

With a higher amount of the oxocluster in the hybrid (1:50 series, with MMA and TFMA), the swelling indexes are generally lower. This can be explained by an enhanced cross-linking, enabled by the greater amount of oxocluster in the 1:50 hybrids, leading to stiffer structures. Accordingly, the hybrids in which the oxocluster is present in a lower amount approximately double their swelling index. Concerning the behaviour of the hybrids in the presence of different solvents, for MMA-based hybrid materials the data evidenced a higher affinity for the less polar solvents and no affinity for water. The TFMA-based hybrid materials show a further increase in the swelling index in ACN and EtOAc, even with low amount of fluorinated monomer (9:1 or 8:2), while negligible changes are observed for EtOH and H_2_O solvents. The hybrids with higher TFMA content, however, exhibit an opposite trend. The hybrid with a 5:5 molar ratio between the two monomers, in particular, shows no further increase for EtOAc and ACN and a relatively higher swelling in water. A possible explanation of such irregular trend is a partial phase separation of the different components, when TFMA increases, leading to a different bulk behaviour. On the contrary, MAPTMS-based materials are swollen in EtOH and H_2_O and are slightly soluble in EtOAc and ACN.

**Figure 4:**
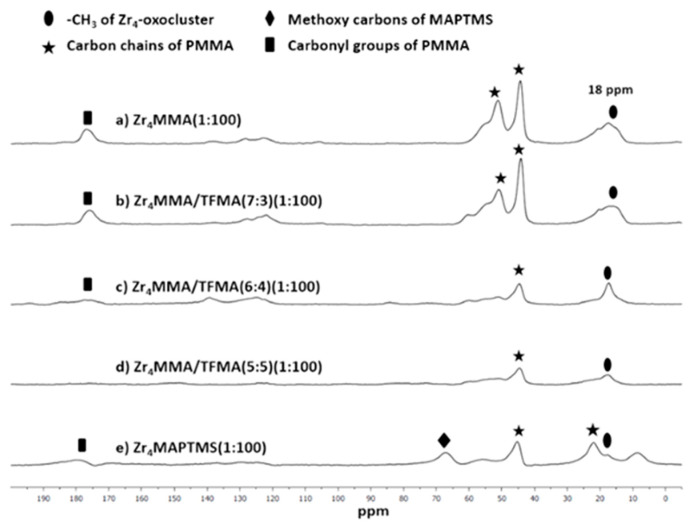
^13^C SS-NMR (400 MHz) of the following hybrids: (**a**) Zr_4_MMA(1:100), (**b**) Zr_4_MMA/TFMA (7:3) (1:100), (**c**) Zr_4_MMA/TFMA (6:4) (1:100), (**d**) Zr_4_MMA/TFMA (5:5) (1:100), (**e**) Zr_4_MAPTMS (1:100).

With the aim of obtaining further information about the hybrids structure, especially on the polymeric matrices, Solid State ^13^C Nuclear Magnetic Resonance Spectroscopy analyses (SS-NMR) were carried out. In Figure 4, the spectra of representative samples are reported.

The signal at about 18 ppm is referred to the methyl groups belonging to Zr_4_ oxocluster and to MMA [14,39]; the signal at 23 ppm, for the Zr_4_MAPTMS(1:100) hybrid, and the peaks at 45 ppm and at 55 ppm, the latter being only present in the Zr_4_MMA (1:100) and Zr_4_MMA/TFMA (7:3) (1:100) hybrids, are related to the main chains of the MMA-[40] and the MAPTMS-[41] based polymers. The broad signal at 68 ppm of the Zr_4_MAPTMS (1:100) hybrid is related to the methoxy carbon atoms of the silane [41]. The carbonyl group of MMA is visible at 178 ppm [39], while the weak signals between 120 and 140 ppm are assigned to residual unreacted olefinic carbon atoms.

### 3.2. Catalytic Tests

The different performances of the synthesised hybrids in the oxidation of methyl p-tolyl sulphide to the corresponding sulfoxide and sulfone were tested at 50 °C in acetonitrile, in the presence of hydrogen peroxide as oxidant, using grinded polymers. In Table 4, yield (%), products selectivity (sulfoxide, SO vs sulphone, SO_2_) and initial rates (R_0_) are listed.

It should be noted that, as already reported, a definite improvement in the catalytic performances can be observed going from the homogeneous conditions (entry 1) to the heterogeneous ones (entries 2–7), with reactions yielding higher conversion (>90%) of the methyl p-tolyl sulphide in 4 h and higher selectivity (>85%) for the sulfone production. Organic polymers, indeed, increase both catalytic activity and stability, owing to their capability to absorb the substrates and to their protection against hydrolytic decomposition of the oxocluster [15]. While MMA-based polymers with different ratio (1:50 or 1:100) display similar activity (entries 2 and 5), and the MMA–TFMA copolymers with 1:50 ratio are rather insensitive to the fluorine content (entries 3 and 4), a much better activity of the fluorinated samples with higher amount of organic fraction (1:100 molar ratio, entries 6 and 7), in terms of initial rate for methyl p-tolyl sulphide conversion and sulfone productivity, was observed. As suggested by their relatively higher swelling index, such improved performance is likely due to an easier access of solvent and substrate to the active sites when a lower cross-linking degree is established. The 1:100 materials were thus screened even under more diluted conditions (with increased amount of solvent), in order to better highlight their behaviour (entries 8–13). The activity of MAPTMS-based hybrids, acting as homogeneous catalysts (see Table 3), was also explored in such conditions (entries 14 and 15). Table 4 evidences that the best samples are the ones with the highest content of TFMA, in particular Zr_4_MMA/TFMA (6:4) (1:100) and Zr_4_MMA/TFMA (5:5) (1:100), which present the best selectivity and the highest initial rate constant (entries 12 and 13). Considering their high affinity shown for all solvents, including water, these polymers can be suitable to promote an optimal accessibility of both substrates and hydrogen peroxide. Within this scenario, a beneficial role of fluorine atoms in the activation of H_2_O_2_ by a network of hydrogen bonds can also be envisaged [26]. On the other hand, the MAPTMS-based hybrids show a lower catalytic activity, as pointed out by a lower yield and much lower selectivity in SO_2_. The partially inorganic matrix is likely responsible for a decreased affinity for the substrate, associated with an even lower affinity for the sulfoxide, which is indeed hardly oxidized to sulphone. As a result, a good selectivity for sulfoxide production can be obtained after 1 h. 

**Figure 5:**
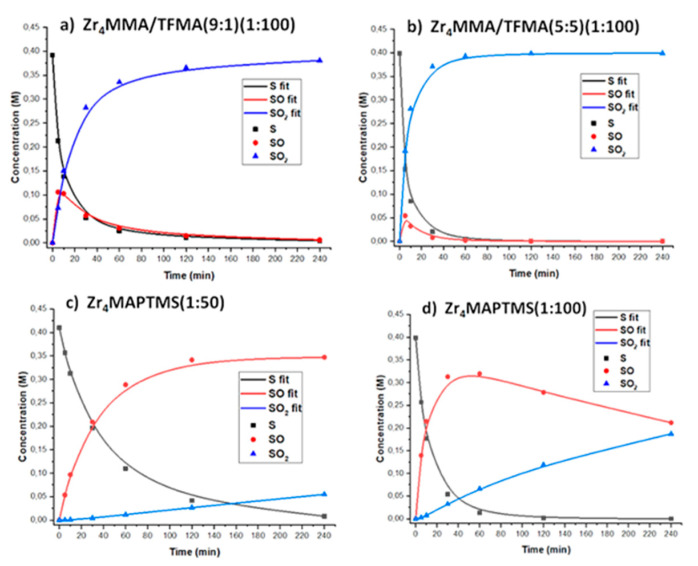
Catalytic performances, including second order kinetic fitting, of (**a**) Zr4MMA/TFMA(9:1) (1:100), (**b**) Zr4MMA/TFMA(5:5) (1:100), (**c**) Zr4MAPTMS(1:50), (**d**) Zr4MAPTMS(1:100). Conditions: 1 mmol sulphide, oxocluster 0.28–0.29 % mol, 2 mmol of H_2_O_2_ in 2.2 mL of ACN, T = 50 °C.

The catalytic performances of these materials are well described by their kinetic traces. In Figure S6 and Figure 5, we compare the evolution of sulphur containing species obtained for the reactions with Zr_4_MMA/TFMA (9:1) (1:100) and Zr_4_MMA/TFMA (5:5) (1:100), the ones with the lowest and the highest content of TFMA, respectively, and between Zr_4_MAPTMS (1:50) and Zr_4_MAPTMS (1:100), characterised by low and high content of MAPTMS, respectively.

The kinetic behaviour is, in all cases, well described by second order kinetics, with reaction rates −d[S]/dt = k[S] and d[SO_2_]/dt = k_2_[SO], where k_1_ and k_2_ are the values for the first and second reaction steps. In Table 5, kinetic constants and the selectivity parameter, reported as the ratio between the kinetic constants (*S* = k_1_/k_2_) are reported.

The hybrid Zr_4_MMA/TFMA (5:5) (1:100) confirms its very promising catalytic activity in terms of rate constant, selectivity and yield, showing a complete conversion of S into SO_2_ at 2h, with *S* = 0.3. This latter value is likely due to a more efficient absorption of sulfoxide, in agreement with an increased swelling, observed for the hybrid, with polar solvents. Regarding Zr_4_MAPTMS (1:50), it is worth noting that the catalytic performances are very different from the other hybrids, with a higher selectivity for the formation of sulfoxide (*S* = 2). On the other hand, with increasing MAPTMS content in the polymeric matrix, i.e., Zr_4_MAPTMS (1:100), an increased yield for sulphone formation can be observed, in agreement with the higher reactivity of the sulfoxide within the polar, less cross-linked material. The parameter *S* is thus lower for the most efficient Zr_4_MMA/TFMA (5:5) (1:100) and higher for Zr_4_MAPTMS (1:50), which represent the two catalysts of choice depending on target oxidised product.

Finally, three catalytic recycles were carried out using the hybrid Zr_4_MMA/TFMA (5:5) (1:100). As reported in Figure S8, the final conversion is constant after the three runs, highlighting a high stability, activity and selectivity even after several catalytic cycles (catalytic turnover number, TON > 1800).

### 3.3. Characterisation of Hybrid Materials after Catalytic Tests

In order to confirm the positive effects of the polymeric matrix against possible changes in both structure and/or composition of the catalytic materials, further analyses were carried out after catalysis. It should be underlined that degradation phenomena could be ascribed either to the polymer matrix or to the oxocluster: (i) leaching of the oxocluster from the polymer matrix; (ii) hydrolysis/condensation or oxidative degradation of the oxoclusters; (iii) oxidation of the polymer matrix [18]. Whereas the first phenomenon is less probable, being the oxocluster covalently linked to the matrix, the others have deserved a closer insight. The studies were focused on Zr_4_MMA (1:100) and Zr_4_MMA/TFMA (5:5) (1:100), to highlight the differences between the stability of the hybrids with and without the fluorinated component, and Zr_4_MAPTMS (1:100). As a first screening, a FT-IR analysis was carried out in order to highlight degradation effects in the polymeric matrix after use, as reported in Figure S3. After the catalysis, the broad band at 3200 cm^−1^ is increased in the Zr_4_MAPTMS (1:100), likely due to additional water absorption by the silica domain. Besides that, we cannot observe any meaningful change in the polymers structure, thus excluding extensive degradation phenomena [9]. An analogue result was obtained by SEM and EDX analyses of Zr_4_MMA/TFMA (5:5) (1:100), which show the retention of the morphology and of the elemental distribution over the material also after catalysis (Figures S12 and S13, and Table S1).

**Figure 6:**
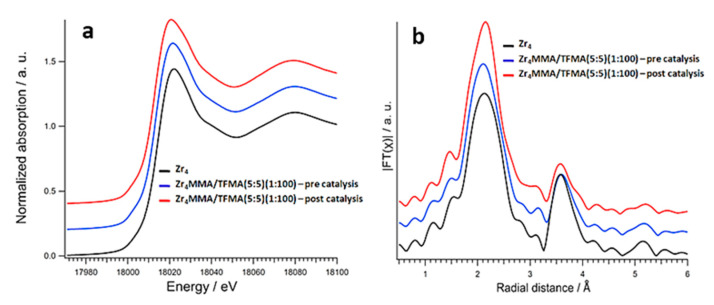
(**a**) Zr K-edge XANES spectra and (**b**) corresponding Fourier transforms of EXAFS curves of Zr4 oxocluster (black lines) and Zr4MMA/TFMA (5:5) (1:100) before (blue lines) and after (red lines) catalysis. Curves are shifted for clarity.

To detail the variations in the inorganic domain after the catalytic reactions, and to verify the structural stability of the oxocluster [42], Zr K-edge X-ray absorption spectra (XAS) were recorded (Figure S7). The properties of these hybrid materials depend on the intermixing, at the molecular level, of the inorganic and organic building blocks. The investigation of the local environment of Zr can indeed provide information on the possible degradation processes after catalysis, as reported in Figure 6.

The Zr K-edge spectrum of the oxocluster Zr_4_ shows a white line peaked at about 18,022 eV, with a less intense shoulder at 18029 eV. These features originate from the splitting of the final *p* states (*s*→*p* transition) and for octahedral centrosymmetric sites becomes very evident. The fact that such a splitting is not so evident in pure Zr_4_ oxocluster indicates that Zr atoms sit in non-centrosymmetric 7- or 8-fold coordination sites, as expected by the oxocluster crystal structure [43]. The spectra of the as prepared hybrid materials strongly resemble that of the reference (Figure S7), suggesting that the oxocluster does not undergo relevant structural changes upon polymerisation with the organic polymers. Only in the case of the Zr_4_MMA (1:100) sample is the width of the white line broader, a clue of an increase in symmetry due to rearrangements around the Zr absorbers [43], while for the diluted MAPTMS-containing sample, the white line intensity is lower. In addition, the presence of an isosbestic point in the absorption spectra (at about 18,035 eV, see Figure S7) proves that the observed spectral changes are only related to changes in coordination around the absorber, and are not related to variations in zirconium content [43]. On the other hand, the spectrum of sample Zr_4_MMA(1:100) does not cross the isosbestic point, indicating a different zirconium concentration in the sample. After catalytic reaction, the hybrids show different stabilities. Interestingly, the Zr_4_MMA (1:100) sample shows a spectrum similar to that of the free oxocluster. This might indicate that, upon polymerisation with MMA, the oxocluster is under tension, while during catalysis its structure can be restored, possibly due to partial loosening of the matrix.

The inorganic component of the compounds prepared with MAPTMS is modified during catalysis.

In the sample 1:50, indeed, the shoulder at 18029 eV increases in intensity, while in the sample 1:100 the splitting becomes clear, accompanied by a sensible decrease in the white line intensity (Figure S7). Owing to the lower degree of polymerization and to the swelling properties of this material, the oxocluster is likely exposed to degradation processes to a higher extent. The fluorinated sample Zr_4_MMA/TFMA (5:5) (1:100) demonstrates, instead, excellent stability, since also after catalysis the spectrum are consistent with an integral Zr_4_ oxocluster structure (Figure 6a). This is also confirmed by comparison of the Fourier transforms of the extended X-ray absorption fine structure (EXAFS) curves (Figure 6b). These fluorinated samples, recovered after catalysis, still present an evident second coordination shell at about 3.53 Å, representing the Zr–Zr distance.

The fitting of the EXAFS curves confirms well what qualitatively indicated by the XANES region, as reported in Table 6.

In accordance with its crystal structure, the pure Zr_4_ oxocluster shows a total Zr–O coordination number of 7.3, divided into two separate oxygen shells at around 2.14 and 2.28 Å. The neighbouring Zr atoms are collected at a distance of 3.53 Å, with a coordination number of 3. By comparison with the results of the fitting of the as-prepared hybrid samples, it can be confirmed that the chemical environment and structure of the inorganic building block are retained upon embedding in the organic matrix. In the case of the sample prepared with MMA, an average higher oxygen coordination number is determined, which is compatible with the broadening of the XANES features, while the number of Zr neighbours decreases. Both Zr–O and Zr–Zr distances grow shorter.

On the other hand, for the catalyst Zr_4_MAPTMS (1:100), the oxygen coordination number is lower than Zr_4_, hinting at the onset of a different degradation process. It should be considered that this effect can also be partially explained in terms of a lower S/N ratio for these samples. The coordination number of zirconium for the sample Zr_4_MMA (1:100), after catalytic testing, falls with respect to the pure Zr_4_ (Zr–Zr 1.4 vs 3.0, respectively) probably due to a degradation process highlighted from different distances Zr–O in the Zr_4_ oxocluster-only and Zr_4_MMA (1:100) hybrid. The XANES spectrum also now intersects the isosbestic point (Figure S7). Overall, this might indicate that the as prepared sample contained a second Zr species that was eliminated during the catalytic process. On the contrary, the TFMA-containing sample confirms again its excellent stability, being well compatible with an integer Zr_4_ model (cfr. both Zr–O and Zr–Zr coordination numbers and bond distances, Table 6). On the other hand, after catalysis, the degradation of the hybrids containing the MAPTMS polymer is clear, especially for the diluted sample, since the first shell coordination number becomes close to 6 while the Zr–Zr are lower in number and at longer distances.

In summary, also the stability of Zr_4_MMA/TFMA (5:5) (1:100), confirms this sample as the best Zr_4_ oxocluster-based catalyst for the oxidation of methyl p-tolyl sulphide to the methyl p-tolyl sulphone. These achievements could be also interesting in the framework of oxydesulfurisation processes, which enable a deep sulphur removal via the efficient conversion of aromatic sulphides to their less soluble sulphones [14].

## 4. Conclusions

A set of novel polymethyl-methacrylate polymers, covalently reinforced by polymerisable Zr_4_ oxoclusters, was prepared and used for the oxidation of methyl p-tolyl sulphide by hydrogen peroxide. By a combination of different investigation tools, based on FT-IR, Raman measurements and ^13^C SS-NMR spectroscopies, it was possible to determine the structure of the hybrids, thus revealing unreacted carbon double bonds eventually present. The swelling measurements were useful to correlate structural properties and reactivity of the different materials, pointing out the better outcome obtained for the MMA/TFMA-based hybrids and for a molar ratio of (1:100) between the polymeric matrix and the oxocluster.

Thermogravimetry highlighted an increased thermal stability for hybrid materials, compared to the blank polymers based on MMA, MAPTMS and MMA/TFMA. The catalytic efficiency of the hybrids was demonstrated in the oxidation of methyl p-tolyl sulphide by H_2_O_2_ in a polar solvent (ACN): the heterogeneous catalysts show an enhanced activity, in terms of reaction yield and selectivity, with respect to the soluble oxoclusters, pointing to a major role of the polymeric matrix in establishing a suitable environment for enhancing catalyst stability and reaction selectivity.

Therefore, it was possible to highlight the increased structural, thermal and chemical stability of the MMA/TFMA based hybrid materials, with particular focus on Zr_4_MMA/TFMA (5:5) (1:100), featuring a molar ratio oxocluster/sum of the monomers of 1:100 and a molar ratio MMA/TFMA of 5:5, which demonstrates a high activity and selectivity for the oxidation of methyl p-tolyl sulphide to the corresponding sulfone and shows an outstanding recyclability after catalytic turnover, as established by EXAFS–XANES analysis.

## Figures and Tables

**Table 1:**
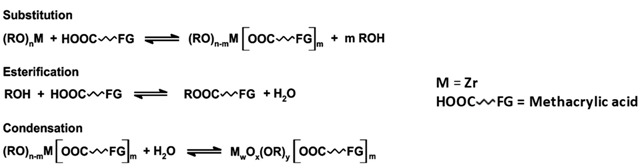
List and names of the hybrid materials based on Zr_4_-oxocluster, prepared with 1:50 or 1:100 oxocluster:monomers molar ratio (benzoyl peroxide was used, in all cases, as radical initiator and the polymerisation was carried out at 85 °C for 1 h) and scheme of the general reaction for the synthesis of the Zr-based oxocluster.

Sample	Monomer (M1)	Co-Monomer (M2)	M1:M2 Molar Ratio	Oxocluster: (M1 + M2) Molar Ratio
Zr_4_MAPTMS (1:50)	MAPTMS	-	-	1:50
Zr_4_MMA (1:50)	MMA		-	
Zr_4_MMA/TFMA (9:1) (1:50)	MMA	TFMA	9:1	
Zr_4_MMA/TFMA (8:2) (1:50)			8:2	
Zr_4_MMA/TFMA (7:3) (1:50)			7:3	
Zr_4_MMA/TFMA (6:4) (1:50)			6:4	
Zr_4_MMA/TFMA (5:5) (1:50)			5:5	
Zr_4_MAPTMS (1:100)	MAPTMS	-	-	1:100
Zr_4_MMA (1:100)	MMA		-	
Zr_4_MMA/TFMA (9:1) (1:100)	MMA	TFMA	9:1	
Zr_4_MMA/TFMA (8:2) (1:100)			8:2	
Zr_4_MMA/TFMA (7:3) (1:100)			7:3	
Zr_4_MMA/TFMA (6:4) (1:100)			6:4	
Zr_4_MMA/TFMA (5:5) (1:100)			5:5	

**Table 2:** Weight residues, based on the molar ratio between the oxocluster and monomers, found by TGA of three synthesised hybrids.

Hybrid	Experimental Residue (% wt.)	Theoretical Residue (% wt.)
Zr_4_MMA (1:100)	3.2	4.3
Zr_4_MMA/TFMA (6:4) (1:100)	8.0	7.4
Zr_4_MAPTMS (1:100)	38.5 ^a^	35.3 ^a^

^a^ 92.4% of the nominal residue is ascribed to SiO_2_.

**Table 3:** Swelling data of the hybrids materials in four different solvents.

Samples	*I_sw_* EtOAc E_T_(30) = 38 [37]	*I_sw_* ACN E_T_(30) = 46 [37]	*I_sw_* EtOH E_T_(30) = 52 [37]	*I_sw_* H_2_O E_T_(30) = 63 [37]
Zr_4_MAPTMS (1:50)	soluble	Soluble	40	25
Zr_4_MMA (1:50)	72	33	29	2
Zr_4_MMA/TFMA (9:1) (1:50)	120	62	29	7
Zr_4_MMA/TFMA (8:2) (1:50)	119	50	24	3
Zr_4_MMA/TFMA (7:3) (1:50)	130	54	30	4
Zr_4_MMA/TFMA (6:4) (1:50)	174	61	29	2
Zr_4_MMA/TFMA (5:5) (1:50)	140	63	22	18
Zr_4_MAPTMS (1:100)	soluble	soluble	29	21
Zr_4_MMA (1:100)	188	84	14	0
Zr_4_MMA/TFMA (9:1) (1:100)	324	116	16	0
Zr_4_MMA/TFMA (8:2) (1:100)	406	109	13	0
Zr_4_MMA/TFMA (7:3) (1:100)	250	151	85	2
Zr_4_MMA/TFMA (6:4) (1:100)	353	162	50	0
Zr_4_MMA/TFMA (5:5) (1:100)	300	102	17	7
PMMA	soluble	soluble	24	6
PMMA/TFMA (6:4)	soluble	soluble	3	1
MAPTMS	soluble	soluble	soluble	soluble

**Table 4:**
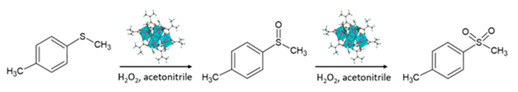
Catalytic performances of the hybrids under more concentrated conditions (0.66 M methyl p-tolyl sulphide, 1.37 M H_2_O_2_ and 1.2 mL of ACN) and less concentrated conditions (0.40 M methyl p-tolyl sulphide, 0.81 M H_2_O_2_ and 2.2 mL of ACN). Substrate: 1 mmol, oxocluster 0.28–0.29% mol, 2 mmol of H_2_O_2_ (from a 35% wt. aqueous solution); T = 50 °C. A general scheme of the catalytic oxidation is reported below.

Entry	ACN Volume (mL)	Catalyst	Yield (%) (1 h)	Yield (%) (4 h)	SO:SO_2_ (1 h)	SO:SO_2_ (4 h)	R_0_ (Ms^−1^)
1	1.2	Zr_4_	49	69	69:31	50:50	4.3 × 10^−5^
2	1.2	Zr_4_MMA (1:50)	86	95	19:81	7:93	1.1 × 10^−4^
3	1.2	Zr_4_MMA/TFMA (9:1) (1:50)	74	90	32:68	15:85	8.1 × 10^−5^
4	1.2	Zr_4_MMA/TFMA (8:2) (1:50)	88	98	19:81	3:97	9.1 × 10^−5^
5	1.2	Zr_4_MMA (1:100)	84	95	21:79	9:91	1.1 × 10^−4^
6	1.2	Zr_4_MMA/TFMA (9:1) (1:100)	93	97	11:89	5:95	1.4 × 10^−4^
7	1.2	Zr_4_MMA/TFMA (8:2) (1:100)	95	99	8:92	2:98	1.6 × 10^−4^
8	2.2	Zr_4_MMA (1:100)	84	95	21:79	9:91	1.1 × 10^−4^
9	2.2	Zr_4_MMA/TFMA (9:1) (1:100)	93	97	11:89	5:95	1.4 × 10^−4^
10	2.2	Zr_4_MMA/TFMA (8:2) (1:100)	95	99	8:92	2:98	1.6 × 10^−4^
11	2.2	Zr_4_MMA/TFMA (7:3) (1:100)	91	92	7:93	8:92	4.1 × 10^−4^
12	2.2	Zr_4_MMA/TFMA (6:4) (1:100)	97	98	1:99	1:99	4.8 × 10^−4^
13	2.2	Zr_4_MMA/TFMA (5:5) (1:100)	99	>99	1:99	0:100	5.0 × 10^−4^
14	2.2	Zr_4_MAPTMS (1:50)	39	55	96:4	87:13	2.0 × 10^−4^
15	2.2	Zr_4_MAPTMS (1:100)	57	76	85:15	47:53	2.8 × 10^−4^

**Table 5:** Kinetic constants and selectivity parameters of Zr_4_MMA (1:100), Zr_4_MMA/TFMA (9:1) (1:100), Zr_4_MMA/TFMA (5:5) (1:100), Zr_4_MAPTMS (1:50), Zr_4_MAPTMS (1:100), calculated under diluted conditions.

Samples	k_1_ (M^−1^s^−1^)	k_2_ (M^−1^s^−1^)	*S* (k_1_/k_2_)
Zr_4_MMA (1:100)	0.0055	0.0099	0.6
Zr_4_MMA/TFMA (9:1) (1:100)	0.003	0.0051	0.6
Zr_4_MMA/TFMA (5:5) (1:100)	0.0045	0.0162	0.3
Zr_4_MAPTMS (1:50)	0.0006	0.0003	2.0
Zr_4_MAPTMS (1:100)	0.002	0.0015	1.3

**Table 6:** Numeric results from fitting the experimental EXAFS spectra with theoretical models.

Sample	Scatterer	N	R (Å)	σ (10^−3^Å)	E_o_ (eV)	*R* Factor
Zr_4_	O1	3.2 ±1.3	2.13 ±0.01	3.9 ±1.0	2.28 ± 1.00	12.2
	O2	4.1 ±0.4	2.27 ±0.01	4.1 ± 1.0		
	Zr	3.0 ±0.8	3.53 ±0.01	4.7 ± 0.5		
Zr_4_MMA (1:100)	O1	3.7 ±1.3	2.12 ±0.02	4.9 ± 1.4	2.92 ± 1.42	23.3
	O2	4.1 ±1.1	2.25 ±0.02	5.1 ±1.6		
	Zr	1.4 ±1.0	3.47 ±0.03	14.3 ± 4.3		
Zr_4_MMA (1:100) after catalysis	O1	2.8 ±1.0	2.12 ±0.02	3.0 ± 0.6	3.84 ± 1.77	20.4
	O2	4.1 ±1.2	2.26 ±0.02	7.0 ± 0.9		
Zr_4_MMA/TFMA (5:5) (1:100)	O1	3.3 ±1.6	2.14 ±0.01	3.0 ± 0.6	3.45 ± 1.27	19.4
	O2	4.1 ±0.4	2.28 ±0.01	5.3 ± 0.5		
	Zr	3.0 ±0.9	3.53 ±0.01	4.7 ± 0.5		
Zr_4_MMA/TFMA (5:5) (1:100) after catalysis	O1	3.5 ±1.3	2.14 ±0.01	3.2 ± 1.1	3.45 ± 1.27	21.3
	O2	4.0 ±1.3	2.27 ±0.01	5.1 ± 2.4		
	Zr	2.8 ±1.4	3.53 ±0.01	7.6 ± 3.4		
Zr_4_MAPTMS (1:50)	O1	3.5 ±1.2	2.13 ±0.01	4.7 ± 0.7	3.70 ± 0.78	9.6
	O2	4.1 ±1.1	2.28 ±0.03	5.3 ± 0.7		
	Zr	1.5 ±0.4	3.56 ±0.01	4.3 ± 1.4		
Zr_4_MAPTMS (1:50) after catalysis	O1	3.5 ±2.0	2.12 ±0.02	2.9 ± 0.7	2.11 ± 1.58	14.9
	O2	4.1 ±0.3	2.25 ±0.02	6.8 ± 1.7		
Zr_4_MAPTMS (1:100)	O1	1.5 ±1.2	2.13 ±0.02	2.3 ± 1.6	5.62 ± 1.13	15.7
	O2	4.0 ±1.3	2.27 ±0.01	6.1 ± 1.4		
	Zr	2.0 ±1.1	3.60 ±0.02	8.7 ± 4.1		
Zr_4_MAPTMS (1:100) after catalysis	O1	2.3 ±1.7	2.12 ±0.03	2.6 ± 0.9	2.33 ± 2.13	26.6
	O2	4.0 ±0.8	2.25 ±0.02	4.2 ± 0.8		
	Zr	0.3 ±1.2	3.58 ±0.02	8.7 ± 2.2		

## Data Availability

Not applicable.

## Supplementary Materials

The following are available online at https://www.mdpi.com/article/10.3390/polym13193268/s1, Figure S1: FT-IR spectra of the samples: (a) Zr_4_MMA (1:50), (b) Zr_4_MAPTMS (1:50), (c) Zr_4_MMA/TFMA (9:1) (1:50), (d) Zr_4_MMA/TFMA (8:2) (1:50)., Figure S2: Full scale of the spectra reported in Figure 2., Figure S3: FT-IR analysis of Zr_4_MMA (1:100), Zr_4_MMA/TFMA (5:5) (1:100) and Zr_4_MAPTMS (1:100) before and after catalysis., Figure S4: Raman mapping of Zr_4_MMA (1:100), collected in a region of 150 x 200 µm and with a power of 8 mW., Figure S5: TGA analyses of the samples: (a) Zr_4_MMA (1:50), (b) Zr_4_MMA/TFMA (8:2) (1:50), 9c) Zr_4_MMA (6:4) (1:50), (d) comparison between three polymers, Zr_4_MMA (1:50), Zr_4_MMA/TFMA (9:1) (1:50) and Zr_4_MMA/TFMA (8:2) (1:50) and PMMA, (e) comparison between three blanks, PMMA, MMA/TFMA (6:4) and MAPTMS, (f) comparison between three polymers, Zr_4_MMA (1:100), Zr_4_MMA/TFMA (6:4) (1:100) and Zr_4_MAPTMS (1:100), whose calculations are reported in Table 2. The TGA were collected with a ramp of 10 °C/min from room temperature (25 °C) up to 900 °C, under air flow., Figure S6: Catalytic tests on the following hybrids: (a) Zr_4_MMA/TFMA (8:2) (1:100), (b) Zr_4_MMA/TFMA (7:3) (1:100), (c) Zr_4_MMA/TFMA (6:4) (1:100). The tests were performed under diluted conditions: 0.40 M methyl p-tolyl sulphide, 0.81 M H_2_O_2_ in 2.2 mL of ACN, Figure S7: Zr K-edge XANES spectra of samples before (left) and after (right) catalysis. The arrows indicate the isosbestic point., Figure S8: Recycles tests on the best synthesised heterogeneous catalysts Zr_4_MMA/TFMA (5:5) (1:100): after the first cycle a conversion of 98% can be observed and a conversion of 96% and 99% after the second and the third catalytic recycles can be pointed out, highlighting the high stability of the samples even after tree catalytic cycles. The tests were performed for 4 h under the same diluted conditions of 0.40 M methyl p-tolyl sulphide, 0.81 M H_2_O_2_ in 2.2 mL of ACN, by recycling the catalyst, Figure S9: SEM-EDX measurements of Zr_4_MAPTMS (1:50), Figure S10: SEM-EDX measurements of Zr_4_MAPTMS (1:100), Figure S11: SEM-EDX measurements of Zr_4_MMA/TFMA (9:1) (1:100), Figure S12: SEM-EDX measurements of Zr_4_MMA/TFMA (5:5) (1:100) before the catalysis, Figure S13: SEM-EDX measurements of Zr_4_MMA/TFMA (5:5) (1:100) after the catalysis, Table S1: Semi-quantitative data from EDX measurements regarding the Zr-content in the Zr_4_MMA/TFMA (5:5) (1:100) before and after the catalytic oxidation.

## Abbreviations

MMA: methylmethacrylate; TFMA, 2,2,2-trifluoroethyl methacrylate; MAPTMS, 3-methacryloxypropyltrimethoxylsilane; BPO, benzoyl peroxide; POSS, polyhedral oligomeric silsesquioxanes; POM, polyoxometalates; PMMA, poly-methylmethacrilate; ACN, acetonitrile; EtOAc, ethyl acetate; EtOH, ethanol; S, sulphide; SO, sulfoxide; SO_2_, sulfone; GC, gas chromatography; TGA, Thermogravimetric analysis; FT-IR, Fourier Transform Infrared; SS-NMR, Solid State Nuclear Magnetic Resonance; XAS, X-ray Absorption Spectroscopy.
