# Supplementary material for: Tuning the Activity of a Hybrid Polymer–Oxocluster Catalyst: A Composition—Selectivity Correlation
Source: Polymers (Basel). 2021 Sep 25;13(19):3268. doi: 10.3390/polym13193268 (PMC8512674; doi:10.3390/polym13193268)
Supplement: Supplementary file 1 [file polymers-13-03268-s001.zip › polymers-1327302-supplementary.pdf]

Supporting information

Giulia Bragaggia[a,b], Anna Beghetto[a], Ferdinando Bassato[a,b], Rudi Reichenbächer[a,c], Paolo Dolcet[a,b,e], Mauro Carraro [a,d], Silvia Gross* [a,b, e]*

[a]Dipartimento di Scienze Chimiche, Università degli Studi di Padova, via Marzolo 1, 35131 Padova, Italy

[b]INSTM, Consorzio Interuniversitario per la Scienza e Tecnologia dei Materiali, via Giusti 9, 50121, Firenze, Italy

[c]Technische Universität Dresden, Helmholtzstr. 10, 01069 Dresden Germany

[d]Istituto per la Tecnologia delle membrane, ITM-CNR via Marzolo 1, I-35131, Padova, Italy

[e] Karlsruher Institut für Technologie (KIT), Institut für Technische Chemie und Polymerchemie, Engesserstr. 20, 76131 Karlsruhe, Germany

*E-mail: mauro.carraro@unipd.it; silvia.gross@unipd.it.

Index

FT-IR analysis 3

Raman mapping 4

TGA analysis 5

Catalytic tests 6

X-ray absorption spectroscopy 7

Recycles tests 7

SEM-EDX measurements 8

FT-IR analysis

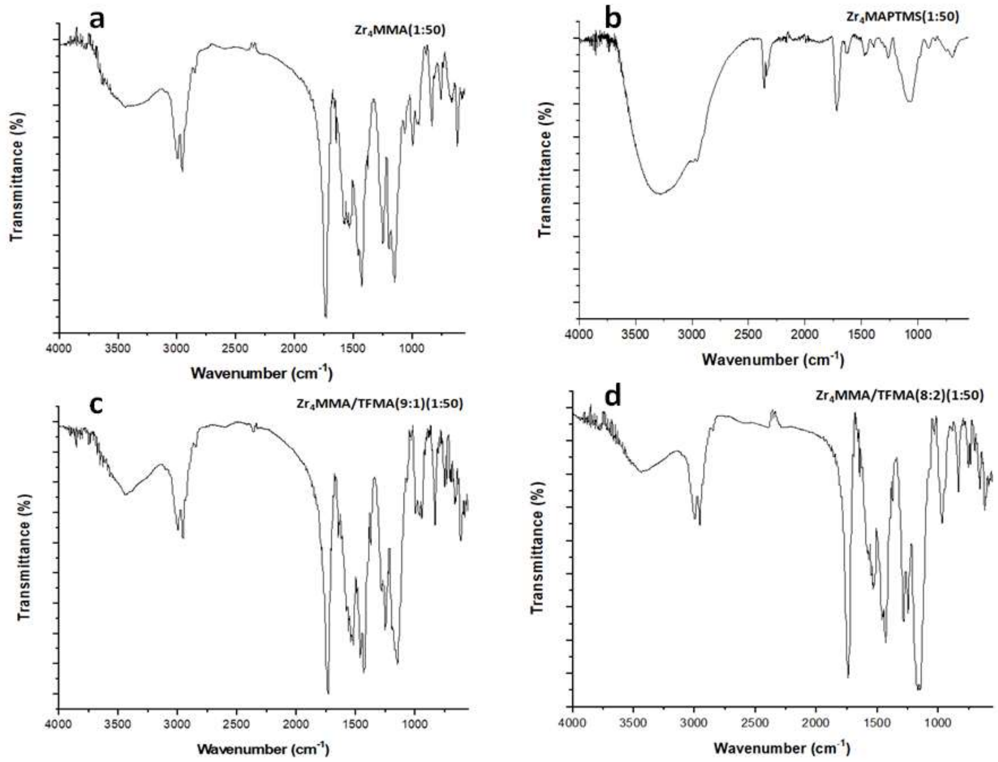

Figure S1. FT-IR spectra of the samples: a) $Zr_4MMA(1:50)$, b) $Zr_4MAPTMS(1:50)$, c) $Zr_4MMA/TFMA(9:1)(1:50)$, d) $Zr_4MMA/TFMA(8:2)(1:50)$.

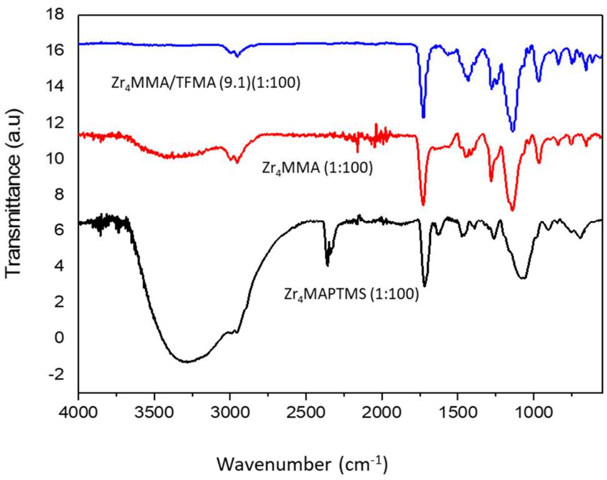

Figure S2. Full scale of the spectra reported in Figure 2.

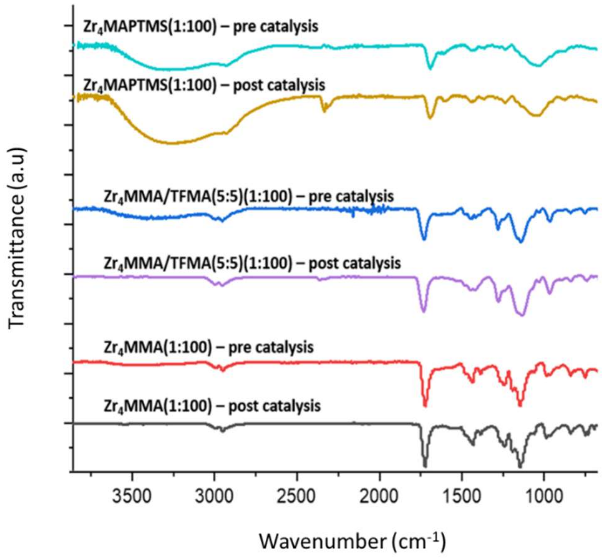

Figure S3. FT-IR analysis of Zr_4MMA(1:100), Zr_4MMA/TFMA(5:5)(1:100) and Zr_4MAPTMS(1:100) before and after catalysis.

Raman mapping

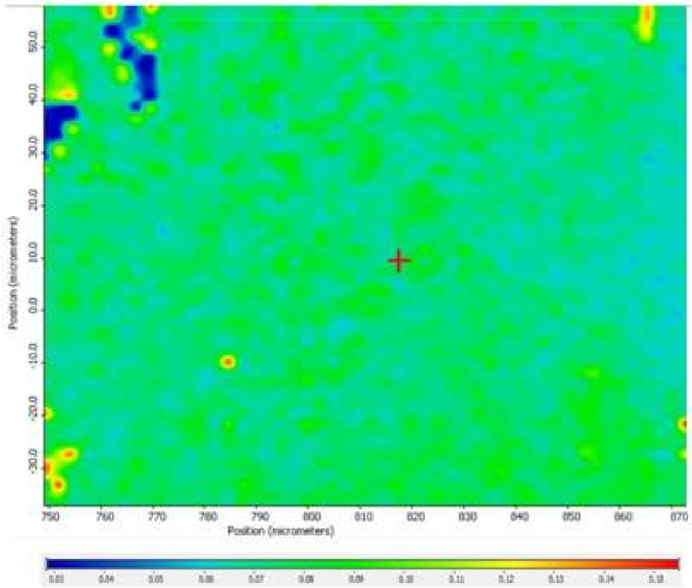

Figure S4. Raman mapping of Zr_4MMA(1:100), collected in a region of 150 x 200 μm and with a power of 8 mW.

TGA analysis

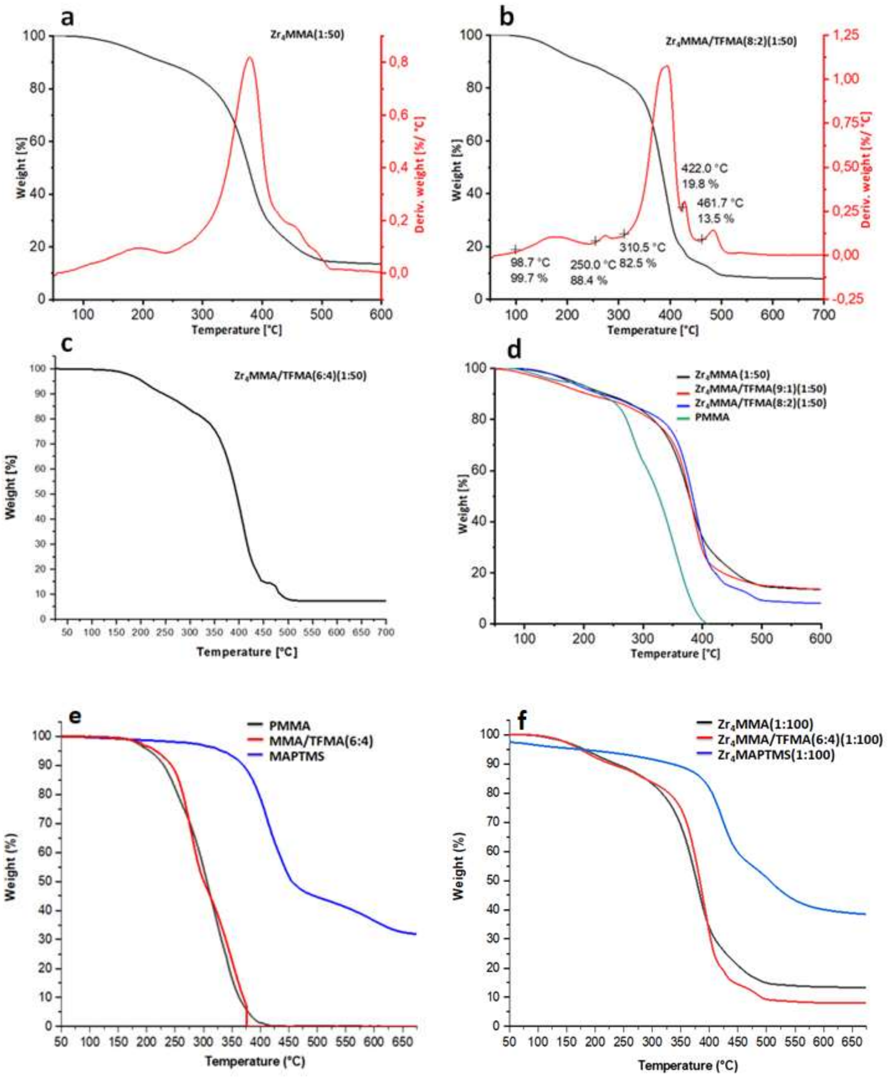

Figure S5. TGA analyses of the samples: a) Zr_4MMA(1:50), b) Zr_4MMA/TFMA(8:2)(1:50), c) Zr_4MMA(6:4)(1:50), d) comparison between three polymers, Zr_4MMA(1:50), Zr_4MMA/TFMA(9:1)(1:50) and Zr_4MMA/TFMA(8:2)(1:50) and PMMA, e) comparison between three blanks, PMMA, MMA/TFMA (6:4) and MAPTMS, f) comparison between three polymers, Zr_4MMA(1:100), Zr_4MMA/TFMA(6:4)(1:100) and Zr_4MAPTMS(1:100), whose calculations are reported in Table 2. The TGA were collected with a ramp of 10°C/min from room temperature (25 °C) up to 900°C, under air flow.

Catalytic tests

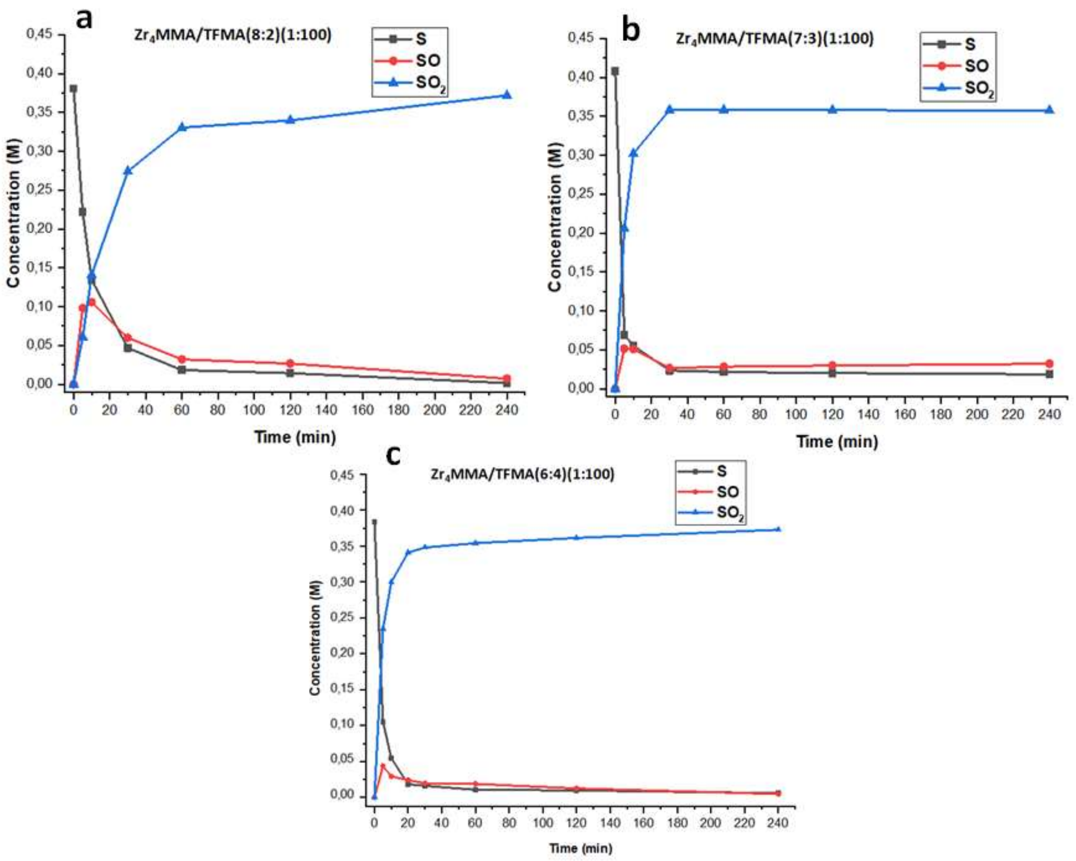

Figure S6. Catalytic tests on the following hybrids: a) Zr_4MMA/TFMA(8:2)(1:100), b) Zr_4MMA/TFMA(7:3)(1:100), c) Zr_4MMA/TFMA(6:4)(1:100). The tests were performed under diluted conditions: 0.40 M sulphide, 0.81 M H_2O_2 in 2.2 mL of ACN.

X-ray absorption spectroscopy

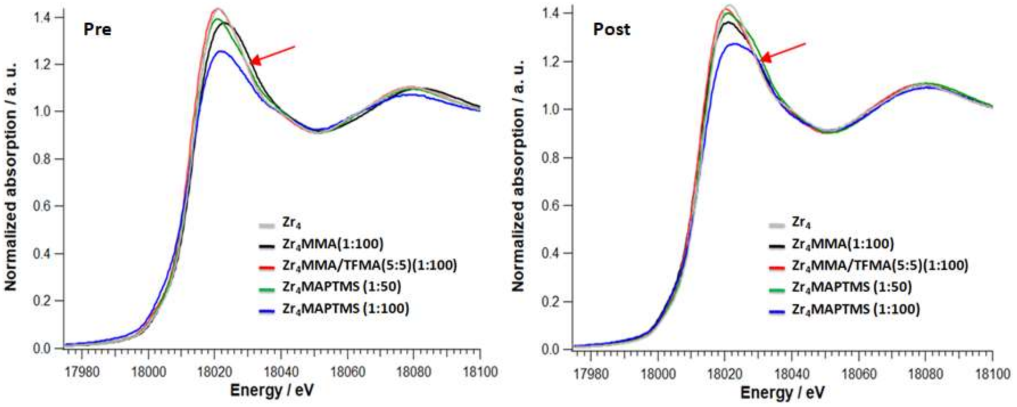

Figure S7. Zr K-edge XANES spectra of samples before (left) and after (right) catalysis. The arrows indicate the isosbestic point.

Recycles tests

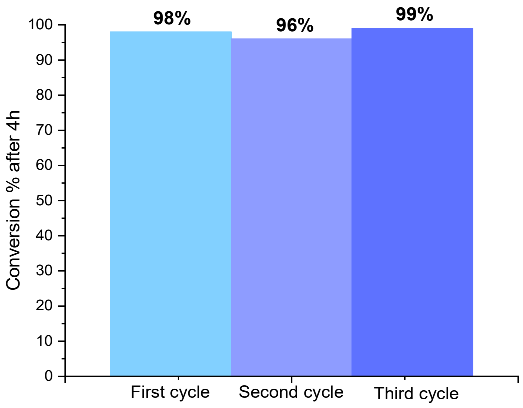

Figure S8. Recycles tests on the best synthesised heterogeneous catalysts Zr_4MMA/TFMA(5:5)(1:100): after the first cycle a conversion of 98% can be observed and a conversion of 96% and 99% after the second and the third catalytic recycles can be pointed out, highlighting the high stability of the samples even after tree catalytic cycles. The tests were performed for 4 hours under the same diluted conditions of 0.40 M sulphide, 0.81 M H_2O_2 in 2.2 mL of ACN, by recycling the catalyst.

SEM-EDX measurements

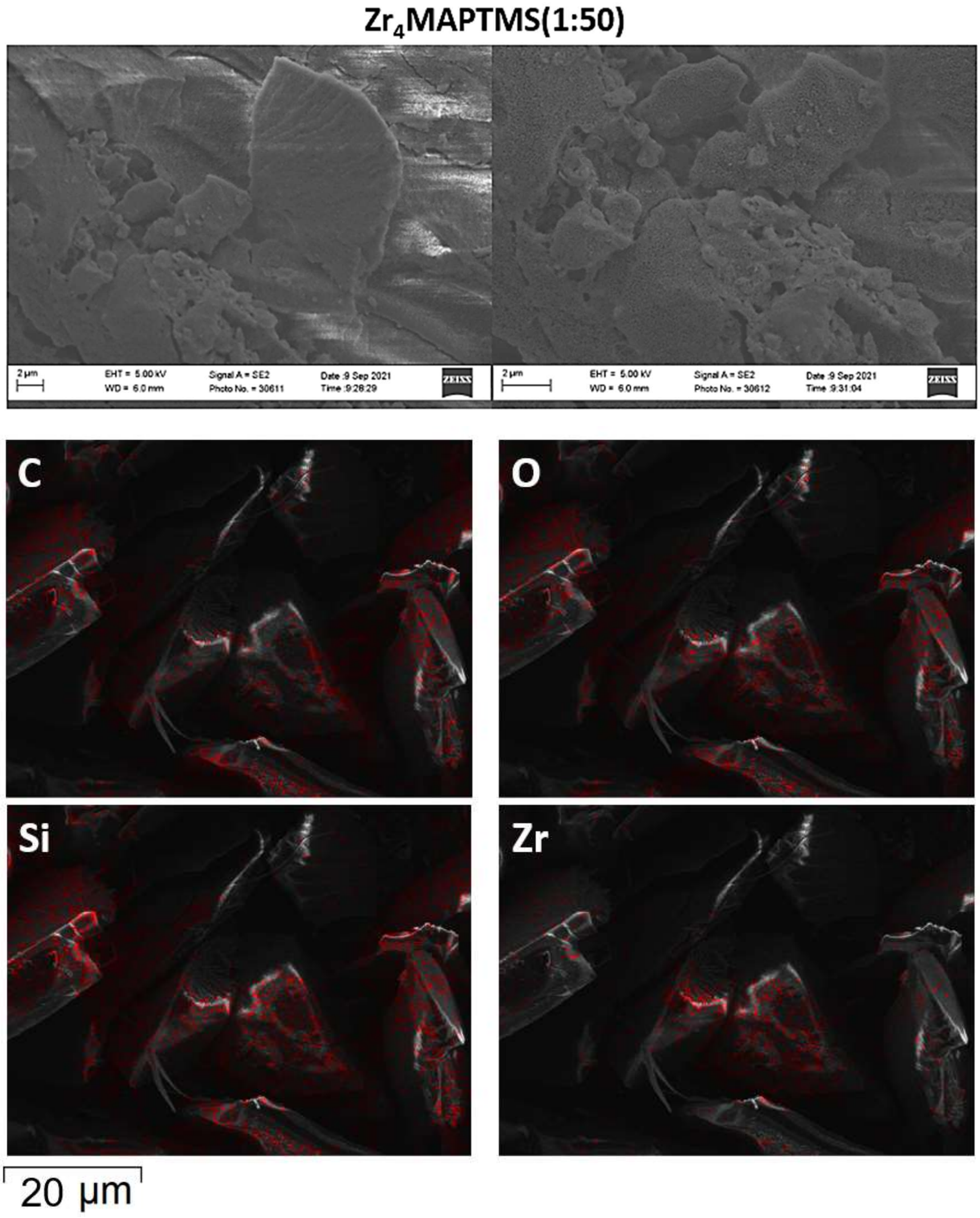

Figure S9: SEM-EDX measurements of Zr_4MAPTMS(1:50).

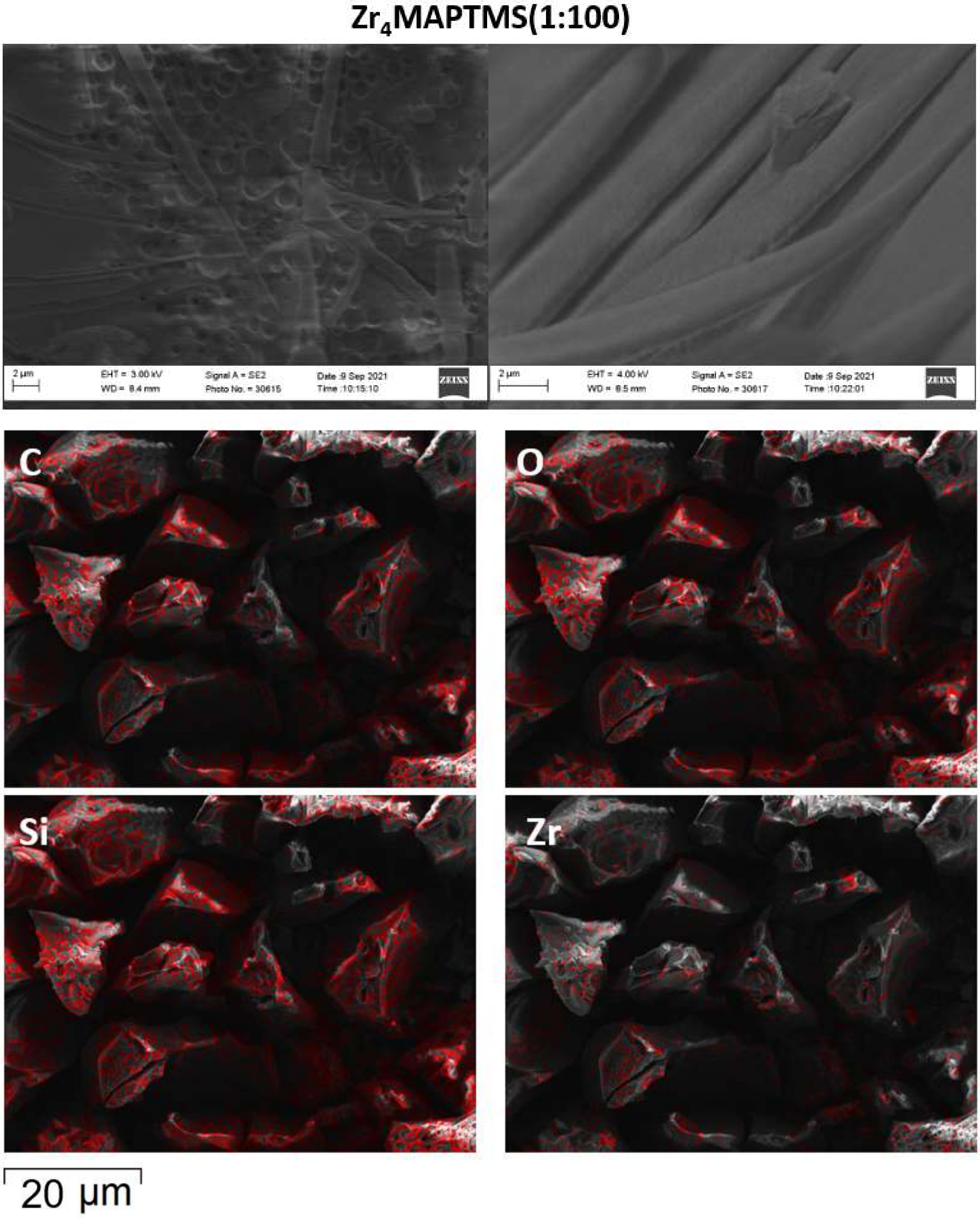

Figure S10: SEM-EDX measurements of Zr_4MAPTMS(1:100).

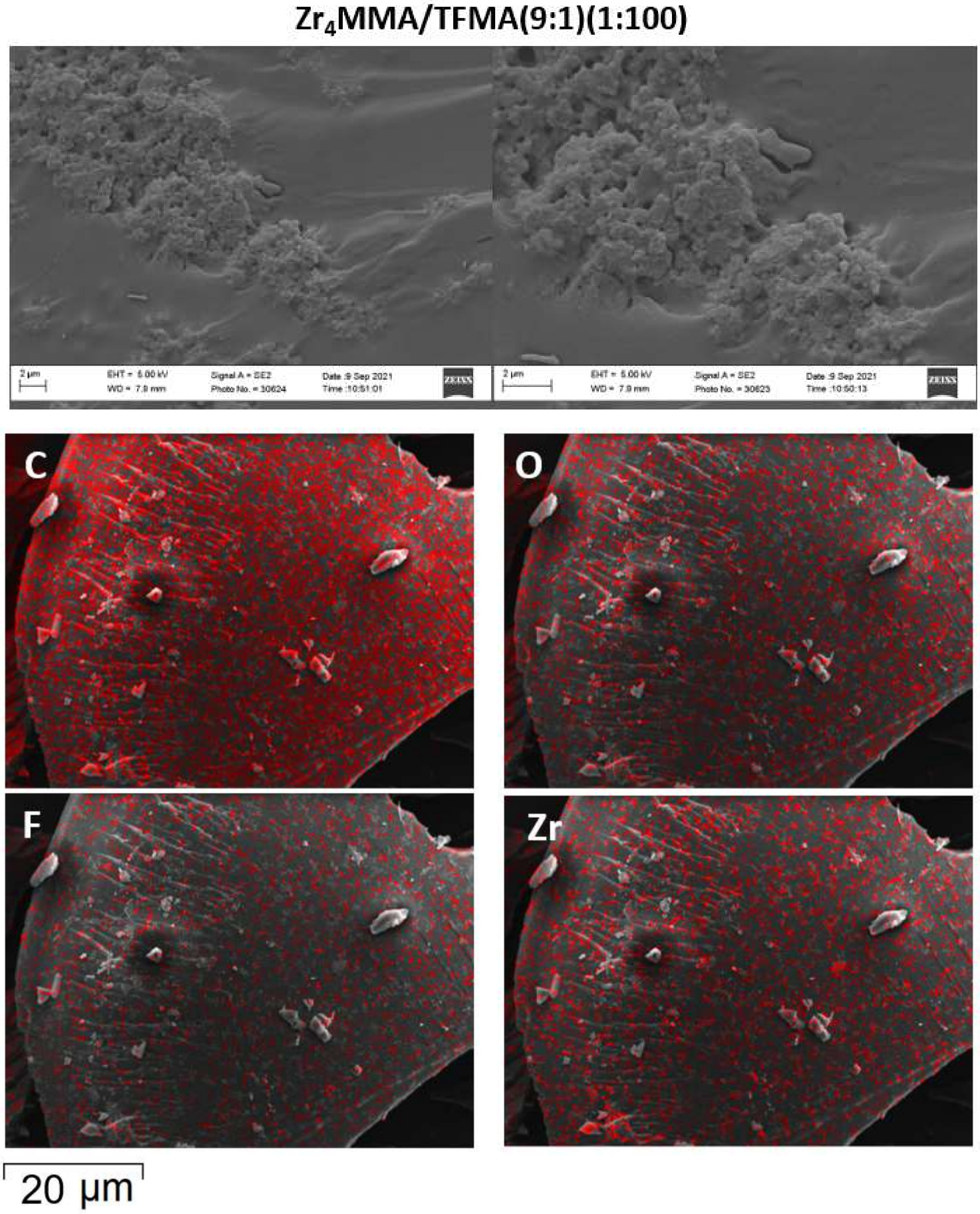

Figure S11: SEM-EDX measurements of Zr_4MMA/TFMA(9:1)(1:100).

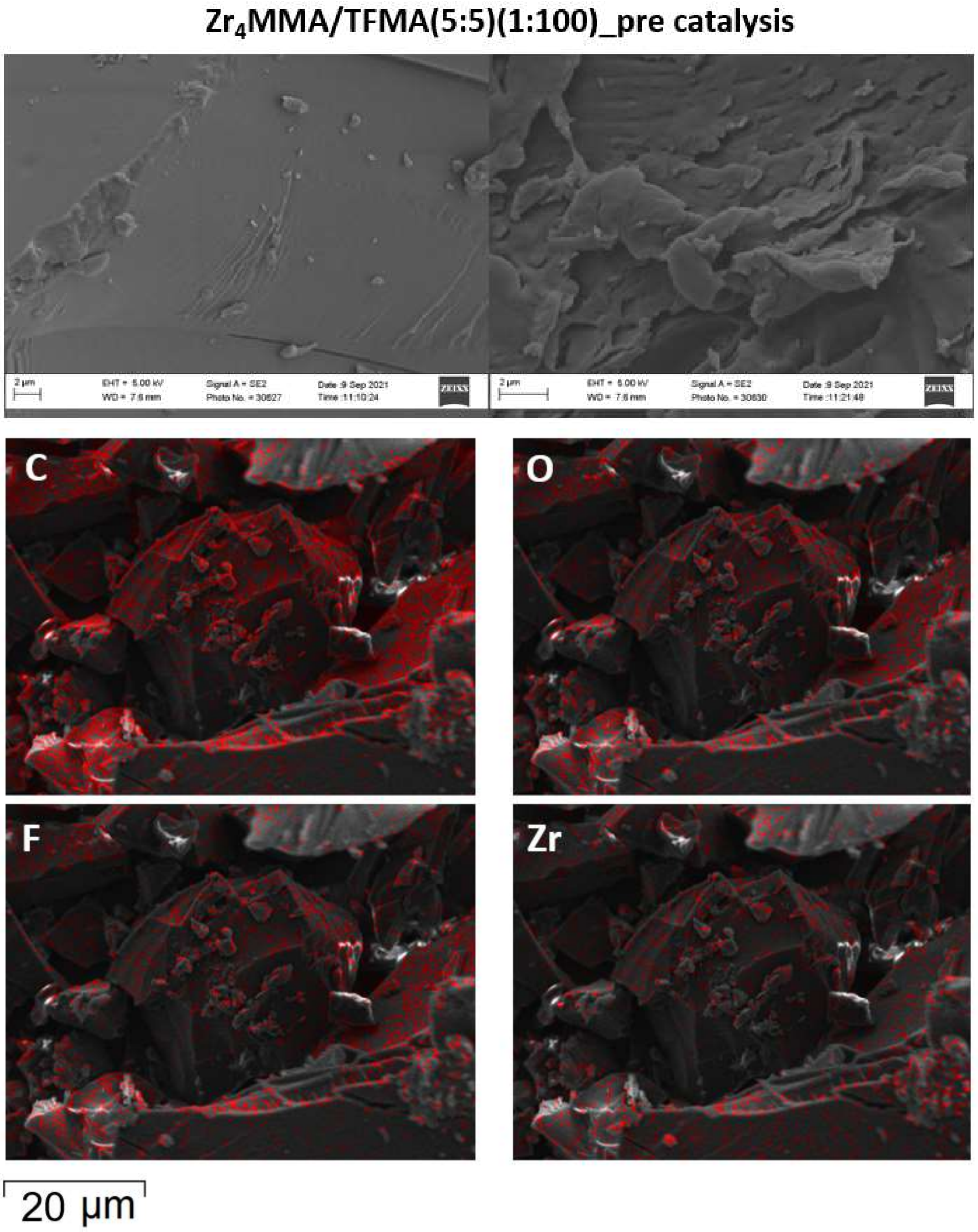

Figure S12: SEM-EDX measurements of Zr_4MMA/TFMA(5:5)(1:100) before the catalysis.

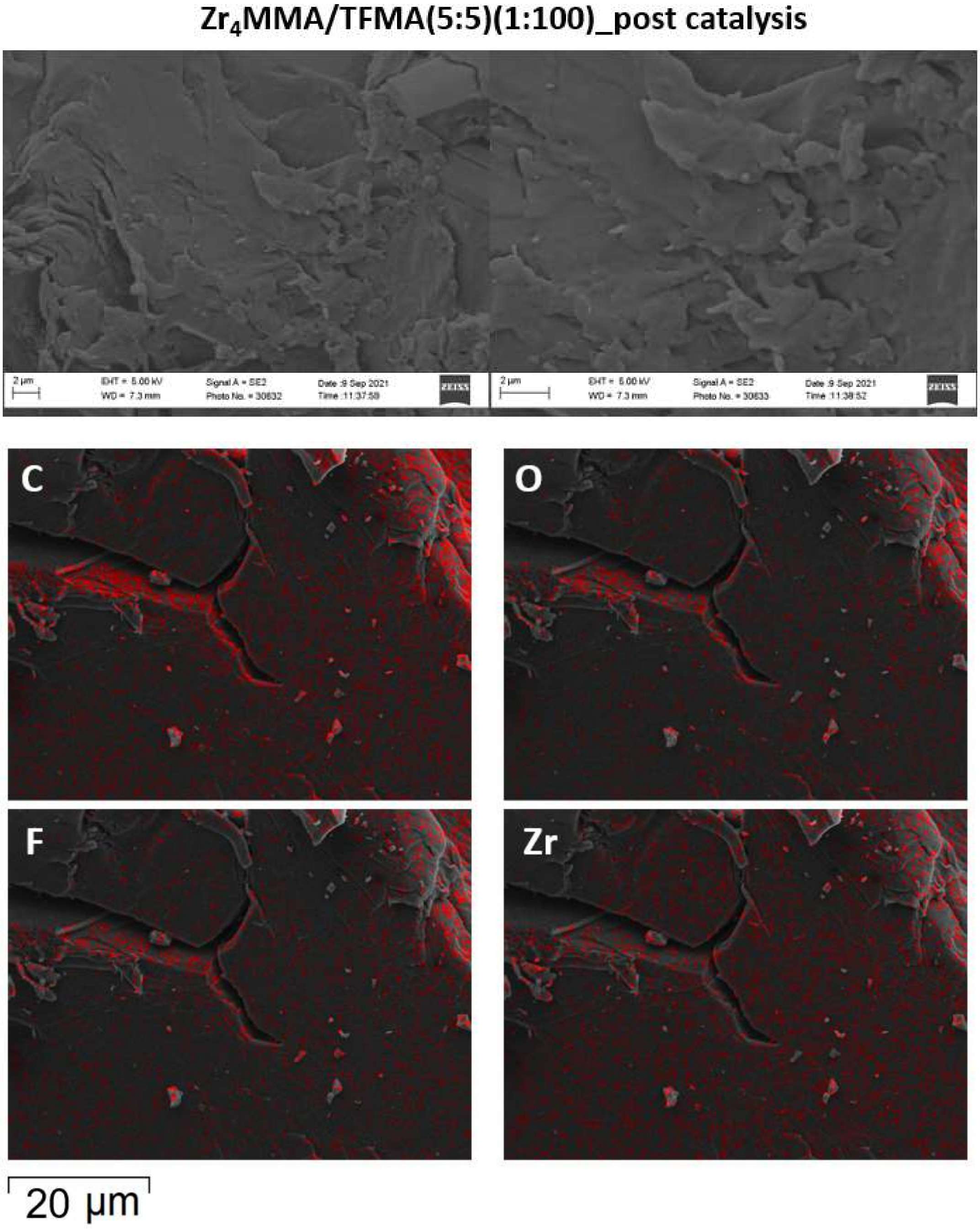

Figure S13: SEM-EDX measurements of Zr_4MMA/TFMA(5:5)(1:100) after the catalysis.

	Zr (% wt.)
Zr4MMA/TFMA(5:5)(1:100)_pre	1,97 ± 0,21
Zr4MMA/TFMA(5:5)(1:100)_post	1,16 ± 0,62

Table S1: Semi-quantitative data from EDX measurements regarding the Zr-content in the Zr_4MMA/TFMA(5:5)(1:100) before and after the catalytic oxidation.
